# Phenothiazine-Based Nanoaggregates: Dual Role in Bioimaging and Stem Cell-Driven Photodynamic Therapy

**DOI:** 10.3390/nano15120894

**Published:** 2025-06-10

**Authors:** Eleonora Calzoni, Alessio Cesaretti, Nicolò Montegiove, Maria Luisa Valicenti, Francesco Morena, Rajneesh Misra, Benedetta Carlotti, Sabata Martino

**Affiliations:** 1 Department of Chemistry, Biology and Biotechnology, University of Perugia, 06100 Perugia, Italy; eleonora.calzoni@unipg.it (E.C.); marialuisa.valicenti@dottorandi.unipg.it (M.L.V.); francesco.morena@unipg.it (F.M.); benedetta.carlotti@unipg.it (B.C.); sabata.martino@unipg.it (S.M.); 2 Centro di Eccellenza Materiali Innovativi Nanostrutturati (CEMIN), University of Perugia, Via Elce di Sotto 8, 06123 Perugia, Italy; 3 Department of Civil and Environmental Engineering, University of Perugia, Via G. Duranti 93, 06125 Perugia, Italy; nicolo.montegiove@unipg.it; 4 Department of Chemistry, Indian Institute of Technology Indore, Indore 453552, India; rajneeshmisra@iiti.ac.in

**Keywords:** nanotechnology, photodynamic therapy, theranostics, bioimaging, mesenchymal stem cells, push–pull compounds, fluorescent probes, photosensitizers, tumor-targeted delivery, aggregation-induced emission

## Abstract

Nanotechnology is transforming contemporary medicine by providing cutting-edge tools for the treatment and diagnosis of complex disorders. Advanced techniques such as bioimaging and photodynamic therapy (PDT) combine early diagnosis and targeted therapy, offering a more precise approach than conventional treatments. However, a significant obstacle for PDT is the need to selectively deliver photosensitizers to disease sites while minimizing systemic side effects. In this context, mesenchymal stem cells have emerged as promising biological carriers due to their natural tropism towards tumors, low immunogenicity, and their ability to overcome biological barriers. In this study, two push–pull compounds, **NPI-PTZ** and **BTZ-PTZ**, phenothiazine derivatives featuring aggregation-induced emission (AIE) abilities, were analyzed. These molecules proved to be excellent fluorescent probes and photosensitizing agents. When administered to human bone marrow-derived multipotent stromal cells (hBM-MSCs) and human adipose multipotent stem cells (hASCs), the compounds were efficiently internalized, maintained a stable fluorescent emission for several days, and showed phototoxicity after irradiation, without inducing major cytotoxic effects under normal conditions. These results highlight the potential of **NPI-PTZ** and **BTZ-PTZ** combined with mesenchymal stem cells as theranostic tools, bridging bioimaging and PDT, and suggest new possibilities for advanced therapeutic approaches in clinical applications.

## 1. Introduction

Nanotechnologies have transformed the landscape of modern medicine, opening up new possibilities for the treatment and monitoring of diseases. Among these, bioimaging and photodynamic therapy (PDT) represent captivating tools for the early diagnosis and targeted therapy of complex diseases such as cancer [1]. However, the effectiveness of these procedures strongly depends on their ability to selectively deliver therapeutic and diagnostic agents to the site of the disease, minimizing systemic side effects. PDT uses a photosensitizer molecule that selectively accumulates in target cells, followed by localized light irradiation of the tumor [2,3,4,5]. By combining specific photosensitizers and focused light, PDT offers greater precision in targeting tumor cells compared to traditional treatments. When the photosensitizer is activated by light, it transfers energy to the oxygen present in tissues, generating reactive oxygen species (ROS), including singlet oxygen (^1^O_2_), superoxide radicals (O_2_^−^), hydroxyl radicals (HO•), and hydrogen peroxide (H_2_O_2_) [1,2]. These ROS trigger a series of biochemical reactions that lead to the destruction of tumor tissues or the induction of apoptosis in the pathological cells [6,7,8]. The detection of light-triggered ROS is therefore crucial to validate the efficacy of a photosensitizer to be used in PDT [9,10,11,12,13]. One of the main obstacles in PDT applications is the limited efficiency of tumor targeting by conventional vehicles, which often fail to overcome biological barriers [14]. This limits the accumulation of photosensitizer compounds at pathological sites, compromising both therapeutic and diagnostic efficacy [15,16]. Furthermore, the development of multifunctional agents capable of combining imaging and therapy remains a significant challenge. In this regard, mesenchymal stem cells have emerged as promising bio-vehicles due to their intrinsic tropism towards tumors and their ability to cross biological barriers [14,16,17]. These properties make them ideal for carrying therapeutic and diagnostic agents, improving their accumulation and distribution in pathological sites. Mesenchymal stem cells show an intrinsic capacity to migrate toward tumors, a phenomenon that is thought to be influenced by the presence of growth factors such as epidermal growth factor (EGF), stromal-derived factor-1 (SDF-1), and platelet-derived growth factor (PDGF) [18,19,20]. In a study conducted with 3D models of breast cancer, human bone marrow mesenchymal stem cells loaded with theranostic polydopamine nanoparticles were shown to effectively integrate into the tumor microenvironment, maintaining viability and showing high antitumor efficacy through chemo-photothermal therapies, superior to conventional methods [21]. Even if the precise mechanisms underlying this behavior are not yet fully understood, abundant experimental evidence has confirmed this property in different tumors, such as brain tumors [22,23,24,25], melanoma [26], and breast cancer [14,27]. Although mesenchymal stem cells show considerable therapeutic potential, certain challenges and long-term safety considerations must be taken into account. These include the potential risk of unwanted differentiation, limited persistence and engraftment in target tissues, and, in specific contexts, the possibility of immune modulation effects that may not always lead to the desired anti-inflammatory outcome. A great deal of effort has gone into better defining and mitigating these aspects to fully harness the clinical benefits of mesenchymal stem cell-based therapies [28,29].

**Chart 1:**
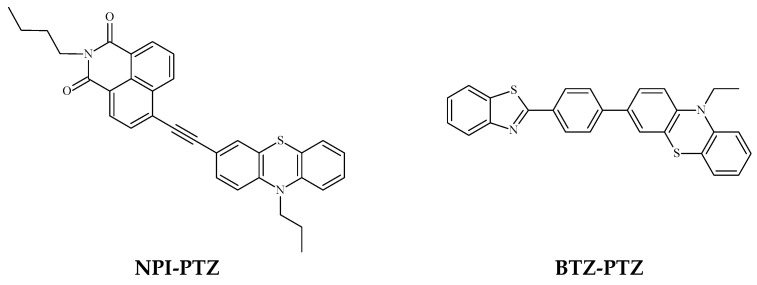
Molecular structures of the investigated compounds.

In this work, two phenothiazine (PTZ) derivatives, namely **NPI-PTZ** and **BTZ-PTZ**, were considered (Chart 1) [2,30,31]. Both **NPI-PTZ** and **BTZ-PTZ** are push–pull systems characterized by the presence of two functional groups with opposite electronic properties: one electron donor and one electron acceptor linked by a conjugated π-bridge. While the push electron-donor portion of both molecules is a PTZ group, the pull electron-acceptor moiety is a naphthalimide (NPI) or a benzothiazole (BTZ) for **NPI-PTZ** and **BTZ-PTZ**, respectively. When these push–pull systems are excited by a light source, an intramolecular charge transfer occurs from the donor to the acceptor through the conjugated system, conveying to the molecules interesting optical properties, such as a marked fluorosolvatochromism or the ability to be excited by two-photon absorption (TPA) [2,30,31,32]. In addition to this, both compounds feature aggregation-induced emission (AIE) in an aqueous environment, which makes them excellent fluorescent probes for biological applications [2,30]. AIE is a photophysical effect in which aggregate species, formed in aqueous dispersions or the solid state, exhibit stronger emission compared to their monomeric counterparts in solution. These two PTZ derivatives have already been extensively characterized and some interesting biological properties have been studied in the context of bioimaging and PDT [2,30,31]. In particular, both push–pull systems were found to undergo intersystem crossing to a triplet state capable of sensitizing the generation of singlet oxygen in solution and, in the case of **BTZ-PTZ**, the formation of ROS even in a cellular environment under light irradiation (λ_exc_ = 390–400 nm), as required in PDT [2,31]. Moreover, PTZ derivatives showed important and sometimes enhanced TPA cross-sections even in the aggregate form, and this would allow for resorting to a NIR excitation in PDT applications [2,30]. Here, **NPI-PTZ** and **BTZ-PTZ** were administered to two different stem cell lines, human bone marrow-derived multipotent stromal cells (hBM-MSCs) and human adipose multipotent stem cells (hASCs), in order to evaluate the internalization of the compounds and their phototoxicity, in light of the possible use of human stem cells as biological vehicles of potent photosensitizers that can be delivered safely without risk of rejection, also allowing their visualization by bioimaging.

## 2. Materials and Methods

### 2.1. Materials

The synthetic procedures for **NPI-PTZ** and **BTZ-PTZ** have previously been reported in [2,31]. The extraction and characterization of hBM-MSCs and hASCs have been widely described. Briefly, hBM-MSCs were derived from bone marrow collected through the washout of femur medullary cavities during primary total hip replacement surgeries performed on adult donors [33]. Similarly, hASCs were extracted from adipose tissue obtained during esthetic procedures [34]. In both instances, the isolation of hBM-MSCs and hASCs occurred from waste samples occasionally and all procedures were conducted with the informed consent of donors, adhering to the principles of the Helsinki Declaration [35]. Dulbecco’s modified Eagle’s medium (DMEM), fetal bovine serum (FBS), Trypsin, and penicillin/streptomycin were purchased from Euroclone (Pero, Italy). Trypan blue powder, 3-(4,5-dimethylthiazol-2-yl)-2,5-diphenyltetrazo- lium bromide (MTT), phalloidin-FITC, Mowiol 4-88, and dimethyl sulfoxide (DMSO) were purchased from Sigma-Aldrich (Saint Louis, MO, USA). 4′,6-diamidino-2-phenylindole (DAPI) was purchased from Vector Laboratories Inc. (Newark, CA, USA).

### 2.2. Human Mesenchymal/Stromal Stem Cell Culture

hBM-MSCs and hASCs were cultured in DMEM containing 10% (*v*/*v*) heat-inactivated FBS and penicillin (10,000 U/mL)/streptomycin (10 mg/mL). The cell concentration was monitored by Trypan blue dye staining, using an automated cell counter (Invitrogen™ Countess™, Thermo Fisher Scientific, Waltham, MA, USA) [36].

### 2.3. MTT Viability Assay and Phototoxicity Assay

A total of 1 × 10^4^ hBM-MSCs and hASCs cells were seeded in Falcon^®^ 96-well clear flat-bottom microplates (Becton, Dickinson and Company, Franklin Lakes, NJ, USA) with 200 μL of DMEM. After 24 h of incubation, 198 μL of fresh DMEM and different dilutions (2 μL) of the **NPI-PTZ** compound stock solution (1 mM in DMSO) or 197 μL of fresh DMEM with different dilutions (3 μL) of the **BTZ-PTZ** compound (0.7 mM in DMSO), respectively, were added into each well to reach concentrations ranging from 10 to 0.01 μM in quadruplicate. A quadruplet was kept as a control (200 μL of DMEM) and another quadruplet was used to take into account the contribution of DMSO (vehicle control = 198 μL of DMEM + 2 μL of DMSO or 197 μL of DMEM + 3 μL of DMSO). After 72 and 96 h of incubation in a humidified atmosphere with 5% CO_2_ at 37 °C, the MTT test was carried out as outlined elsewhere [37]. Cell viability was expressed as the optical density percentage, determined at 570 nm using a microplate reader (Beckman Coulter DTX880, Beckman Coulter, Inc., Brea, CA, USA), in treated cells compared with vehicle controls, assuming that the absorbance of controls was 100% (absorbance of treated wells/absorbance of control wells × 100). All measurements were performed in two independent experiments. The phototoxicity of **NPI-PTZ** and **BTZ-PTZ** compounds was tested by treating the cells in the same way and proceeding with a photoexposure of 25 min with an LED chamber (λ_exc_ = 390–400 nm) producing a power of about 1.7 mW/cm^2^. Also, in this case, the cell viability was determined through the MTT cell viability assay.

### 2.4. Intracellular ROS Production

Intracellular ROS production was evaluated through the H_2_DCFDA method, which has been widely described elsewhere [2,38]. H_2_DCFDA is a widely adopted probe for the detection of oxidative stress in cells, by virtue of the high sensitivity of fluorescence-based analytical methods [39,40]. In particular, 5 × 10^3^ cells/well of hBM-MSCs and hASCs were seeded in Corning^®^ 96-well black round-bottom polystyrene microplates (Corning Incorporated, Corning, NY, USA) in 200 µL of DMEM culture medium. The following day, the medium was replaced with fresh DMEM containing different concentrations of the **BTZ-PTZ** and **NPI-PTZ** compounds ranging from 10 to 0.01 μM. A quadruplet was kept as vehicle control to take into account the effect of DMSO (197 µL of DMEM + 3 µL of DMSO or 198 μL of DMEM + 2 μL of DMSO, respectively), and another quadruplet was used to monitor the possible contribution of the compounds’ autofluorescence. Cells were photoexposed for 25 min in the LED chamber (λ_exc_ = 390–400 nm) with a power of about 1.7 mW/cm^2^. After 30 min, cells were washed with 100 µL of PBS and incubated with 10 μM H_2_DCFDA for 60 min, in a humidified atmosphere, at 37 °C and 5% CO_2_. Wells were first washed with 100 µL of PBS and then filled with 200 µL of the same buffer. The fluorescence intensity, arising from DCF oxidized under the action of ROS, was measured using a microplate reader (Beckman Coulter DTX880, Beckman Coulter, Inc., Brea, CA, USA) by exciting the samples at 485 nm and reading the emission at 530 nm. Simultaneously, the viability of cells treated and photoexposed under the same experimental conditions was assessed through the MTT assay performed on another Falcon^®^ 96-well clear flat-bottom microplate (Becton, Dickinson and Company, Franklin Lakes, NJ, USA). Data were thus expressed as the percentage of DCF fluorescence intensity relative to vehicle controls and normalized to cell viability. All measurements were performed in quadruplicate in two independent experiments.

### 2.5. Trypan Blue Exclusion Assay

Cell growth was monitored using Trypan blue dye staining with an automated cell counter (Invitrogen™ Countess™, Thermo Fisher Scientific, Waltham, MA, USA). Specifically, 5 × 10^4^ hBM-MSCs and 2 × 10^4^ hASCs were seeded into Falcon^®^ 24-well clear flat-bottom plates (Becton, Dickinson and Company, Franklin Lakes, NJ, USA). After 24 h, the culture medium was replaced with 400 µL of fresh DMEM containing concentrations of **NPI-PTZ** and **BTZ-PTZ** of 1 and 10 μM, obtained as previously described in Section 2.3. Cell growth was assessed at 3, 6, 12, 24, 48, and 72 h, both in the dark and after 25 min of photoexposure, using a 0.04% Trypan blue solution.

### 2.6. Fluorescence Microscopy Analysis

Fluorescence microscopy was used both to evaluate the fluorescence emission of the compounds before and after photoexposure and to evaluate their fluorescence stability over time. A total of 1.5 × 10^3^ hBM-MSCs and hASCs were seeded on round glass coverslips previously sterilized via 30 s of immersion in 70% ethanol, rinsed with sterile phosphate-buffered saline (PBS), and placed in a Falcon^®^ 24-well clear flat-bottom multiwell cell culture plate (Becton, Dickinson and Company, Franklin Lakes, NJ, USA). The cells were then incubated for 24 h in a humidified atmosphere with 5% CO_2_ at 37 °C with 400 μL of fresh DMEM. After 24 h of incubation, either 400 μL of an **NPI-PTZ** compound solution diluted in DMEM at a final concentration of 10 μM or 400 μL of a DMEM solution containing **BTZ-PTZ** and a nucleus fluorescent marker (C5) [41,42], both at a concentration of 10 μM, were administered to the cells and incubated for 2 h under standard conditions. The cells on round glass coverslips were then rinsed twice with PBS and fixed in 4% paraformaldehyde for 20 min at room temperature. After washing with PBS, some samples were treated for 20 min at room temperature with Phalloidin (Alexa Fluor 488 Phalloidin, Sigma-Aldrich, Saint Louis, MO, USA) for F-Actin staining. Samples containing **NPI-PTZ** were then mounted, staining nuclei with VECTASHIELD Vibrance^®^ Antifade Mounting Medium containing DAPI (Vector Laboratories, Inc., Newark, CA, USA), while samples treated with **BTZ-PTZ**, whose nuclei had already been stained with C5, were mounted with Mowiol 4-88 (Sigma-Aldrich, Saint Louis, MO, USA). To evaluate the effect of photoexposure, before being mounted, half of the treated samples were photoexposed to light for 25 min in the LED chamber (λ_exc_ = 390–400 nm), incubated for 2 h, and subsequently mounted. Image acquisition was performed using a fluorescence microscope (Eclipse TE2000-S, Nikon, Tokyo, Japan) equipped with the F-View II FireWire camera (Olympus Soft Imaging Solutions GmbH, Münster, Germany) and through the use of CellF Imaging Software (Olympus Soft Imaging Solutions GmbH, Münster, Germany). To assess the fluorescence stability over time of the two compounds in a cellular environment without photoexposure, fluorescence images were acquired at 24, 48, 72, 96, 120, and 144 h after treatment with the two compounds. In this case, all the cells were initially treated as previously described and then, after waiting for a specified time, they were fixed with 4% paraformaldehyde and subsequently mounted.

#### 2.6.1. Fluorescence Intensity Quantification over Time

The fluorescence intensity of **NPI-PTZ** and **BTZ-PTZ** over time in hBM-MSCs and hASCs as well as the fluorescence intensity of stem-cell nuclei treated with the two compounds in dark conditions and after 25 min of photoexposure in an LED chamber (λ_exc_ = 390–400 nm and a power of 1.7 mW/cm^2^) were quantified using ImageJ software (version 1.54p). For each cell sample, 5 stained cells were analyzed. Individual cells were selected as regions of interest (ROIs) using the selection tools. The integrated density, area, and mean gray value were extracted from each ROI. To correct background fluorescence, five measurements were taken from non-cellular regions in each image, and the mean background intensity was calculated [43]. The corrected total cell fluorescence (CTCF) of both compounds over time and cellular nuclei in dark conditions and after photoexposure was determined using the following formula [44,45,46]:

$$\mathrm{CTCF}=\mathrm{IntDen}-\left(A\;\times {\;\mathrm{BG}}_{\mathrm{mean}}\right)$$

where

CTCF = corrected total cell fluorescence;
IntDen = cell fluorescence integrated density;
A = area of the selected cell;
BG_mean_ = mean fluorescence of the background readings (calculated as the mean of five different ROIs).

Images were processed using identical settings for thresholding and analysis across all time points. The obtained CTCF values were averaged across analyzed cells, transformed into LogCTCF by using the base-10 logarithm, and plotted to illustrate the relative fluorescence intensity variations over time.

#### 2.6.2. Evaluation of Maximum Feret’s Diameter of Nuclei

The measurement of the maximum Feret’s diameter of cellular nuclei was performed using the ImageJ software (version 1.54p) [47,48]. For each sample, 10 different nuclei were measured in both hBM-MSCs and hASCs treated with **NPI-PTZ** and **BTZ-PTZ** under dark conditions and after 25 min of photoexposure in an LED chamber (λ_exc_ = 390–400 nm and a power of 1.7 mW/cm^2^). The mean values of the maximum Feret’s diameter expressed in µm were compared between non-photoexposed and photoexposed cells for each cell type and treatment.

### 2.7. Statistical Analysis

The analyses performed in this study are presented as mean values ± standard deviation (SD). A one-way ANOVA followed by Tukey’s multiple comparisons test was used to assess the significance of differences in sample means. A significance level of *p* < 0.05 was set for all analyses. Statistical tests were conducted using GraphPad Prism 9.0.0 for Windows (GraphPad Software, San Diego, CA, USA).

## 3. Results

### 3.1. Analysis of Cell Growth Inhibition and IC_50_ Determination of Phototoxic Compounds

**Figure 1:**
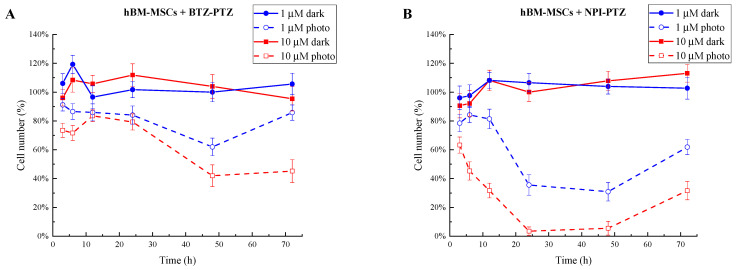

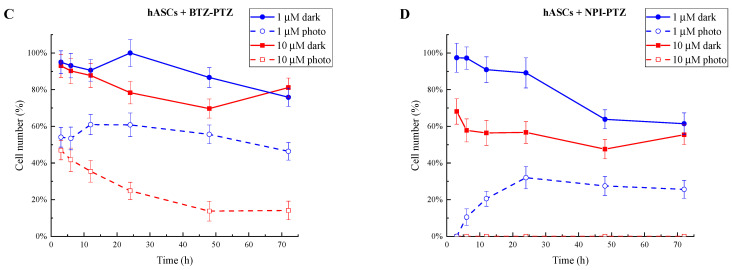
Stem cell viability of hBM-MSCs (upper panels, (**A**,**B**)) and hASCs (lower panels, (**C**,**D**)) assessed at 3, 6, 12, 24, 48, and 72 h of incubation with **BTZ-PTZ** and **NPI-PTZ** used at two distinct concentrations (1 and 10 µM in blue and red, respectively). Cells were either kept in the dark (dark, solid line) or photoexposed for 25 min in the LED chamber (λ_exc_ = 390–400 nm and a power of 1.7 mW/cm^2^) (photo, dashed line). Values are reported as the mean of three independent experiments ± SD; 100% corresponds to control mean values.

Stem cells were treated with the two compounds and their viability was monitored over time for as long as 72 h to evaluate the toxicity of the two molecules in the absence of light and after photoirradiation. Growth curves, reported as the percentage of live cells relative to untreated control cells as a function of time (Figure 1), were thus obtained by employing the Trypan blue method.

Growth curves show that in the absence of photoactivation (solid lines in Figure 1), both **BTZ-PTZ** concentrations used (1 and 10 µM) did not induce significant cytotoxic effects on hBM-MSCs, with cell viability values in the presence of both compounds around 100% relative to untreated control cells up to 72 h. Conversely, in the case of hASCs, a reduction in cell viability was recorded, with minimum values of about 70% and 50%, reached 48 h after the treatment with 10 µM of **BTZ-PTZ** and **NPI-PTZ**, respectively. On the other hand, photoexposure resulted in a significant reduction in cell viability, with an effect strictly dependent on the dose, time, and compound used. For **BTZ-PTZ**, hBM-MSCs (Figure 1A) treated with 10 µM (red dashed line) showed a reduction in cell viability below 80% after 3 h, reaching a minimum of approximately 40% at 48 h, while a lower concentration (1 µM, blue dashed line) induced a similar trend with slightly higher live cell percentages. **NPI-PTZ** showed similar trends but higher phototoxicity: in hBM-MSCs (Figure 1B), a concentration of 1 µM (blue dashed line) reduced cell viability to below 40% within 24 h, though it ultimately increased again after a longer time period, while 10 µM (red dashed line) caused a decrease in cell viability below 10% during the first 24 h, with a final enhancement at 72 h. This behavior might be rationalized by considering that surviving cells could increase their population over a long timeframe, contributing to the ultimate growth of the curve. hASCs showed greater sensitivity to the same treatments. In the case of **BTZ-PTZ** (Figure 1C), cell viability dropped below 50% after 3 h and further reduced to 15% at 72 h when used at 10 µM (red dashed line), while an initial drop around 55% decreasing below 50% at 72 h was measured with a 1 µM concentration (blue dashed line). To understand this complex behavior, the slight effect of dark cytotoxicity, adding to immediate phototoxicity, has to be considered in the long run. In particular, the further reduction detected at the longest incubation times could be related to the modest dark toxicity brought about by **BTZ-PTZ**. Finally, the treatment of hASCs with both concentrations of **NPI-PTZ** (Figure 1D) was responsible for the almost complete disappearance of the cell population, as revealed at the first data collection point, i.e., 3 h; then, cell viability remained almost zero at the highest concentration (red dashed line), while it grew back to about 30% after 24 h and decreased again after a longer time period with 1 µM (blue dashed line). In this latter case, after most cells were promptly suppressed, the surviving ones could start to undergo reproduction, thus increasing their number, which would eventually be reduced again because of the small, albeit non-negligible, dark toxicity exerted by **NPI-PTZ** as the incubation time increased.

The MTT assay was used to assess the dark cytotoxic effect 72 and 96 h after the treatment with the two compounds at a concentration ranging from 0.01 to 10 µM. The complete data from the MTT assay are reported in the Supporting Information (Figures S1 and S2). Neither **BTZ-PTZ** nor **NPI-PTZ** showed a significant effect on the viability of hBM-MSCs after 72 and 96 h of incubation in the dark (Figure S1A,B), with cell viability remaining above 90% even at high concentrations (10 µM). Conversely, in hASCs (Figure S1C,D), the highest concentration of **BTZ-PTZ** reduced cell viability in a time-dependent manner, reaching levels around 85% after 72 h and just below 70% after 96 h.

Subsequently, the phototoxic effect of the two sensitizers was also evaluated by irradiating the treated cells in an LED chamber (λ_exc_ = 390–400 nm and 1.7 mW/cm^2^) for either 12 min and 30 s or 25 min and later performing the MTT test after waiting 72 h (Figure S2). Upon photoexposure, the antiproliferative activity of both compounds increased drastically when used at the highest concentration of 10 µM: in hBM-MSCs (Figure S2A,B), **NPI-PTZ** reduced viability to 50% after 12 min and 30 s of photoexposure and less than 20% after 25 min, whereas in hASCs (Figure S2C,D), cell viability dropped to around 35% with a reduced irradiation time and to below 20% when doubling the time of photoexposure. The same concentration of **BTZ-PTZ** caused a still important but reduced effect, with the viability of hBM-MSCs photoexposed for the longest irradiation time decreasing below 40% and that of hASCs decreasing to around 25%. These data were used to calculate the IC_50_ values (Table 1), analyzing the cellular responses to **BTZ-PTZ** and **NPI-PTZ** after photoexposure as a function of concentration.

Quantitative data from the IC_50_ values confirm the above-mentioned results. For **BTZ-PTZ**, the IC_50_ regarding hBM-MSCs was 8.1 ± 0.9 µM after 25 min of photoirradiation, while this value was significantly lower regarding hASCs (2.2 ± 0.2 µM), indicating a higher susceptibility of the latter cell line to the treatment. **NPI-PTZ**, meanwhile, exhibited an even higher phototoxicity, with IC_50_ values of 6.3 ± 0.6 µM for hBM-MSCs and 1.3 ± 0.1 µM for hASCs, respectively.

### 3.2. Evaluation of Intracellular ROS Production

**Figure 2:**
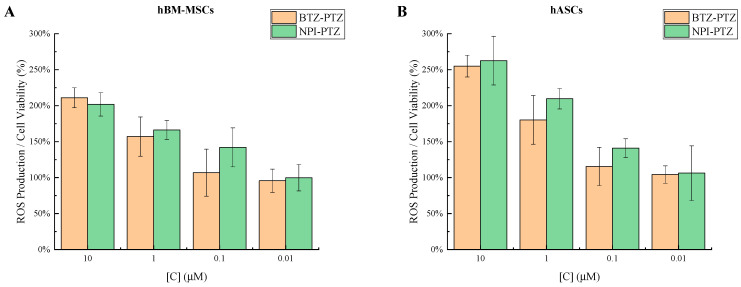
ROS production normalized to cell viability of different concentrations of compounds **BTZ-PTZ** and **NPI-PTZ** in hBM-MSCs (**A**) and hASCs (**B**) photoexposed for 25′ in an LED chamber (λ_exc_ = 390–400 nm and a power of 1.7 mW/cm^2^). Values are expressed as the mean of two independent experiments of four replicas each ± SD; 100% corresponds to control mean values.

In order to assign the phototoxic effect exerted by the two compounds on both cell lines to the expected production of ROS, their levels were evaluated through the H_2_DCFDA method. Significant fluorescence signals, due to the oxidation of DCF by ROS, were revealed for the highest concentrations (10 and 1 µM) of both **BTZ-PTZ** and **NPI-PTZ** in cells exposed for 25 min in the LED chamber. These values were normalized to the cell viability measured through the MTT assay, thus allowing the production of ROS in treated cells to be compared with that in untreated samples (Figure 2).

A concentration of 10 µM caused the levels of intracellular ROS to rise above 200% in hBM-MSCs relative to untreated cells, and to around 250% in hASCs, in line with the greater phototoxicity found in the latter cells through the other tests. These values were also found to be significantly higher than 100% in cells treated with 1 µM **BTZ-PTZ** and **NPI-PTZ**, again confirming how even this lower concentration could still compromise cell viability after photoirradiation. No significant differences were measured for the level of ROS produced by the two photoexcited compounds under the same concentration conditions.

### 3.3. Fluorescence Microscopy

**Figure 3:**
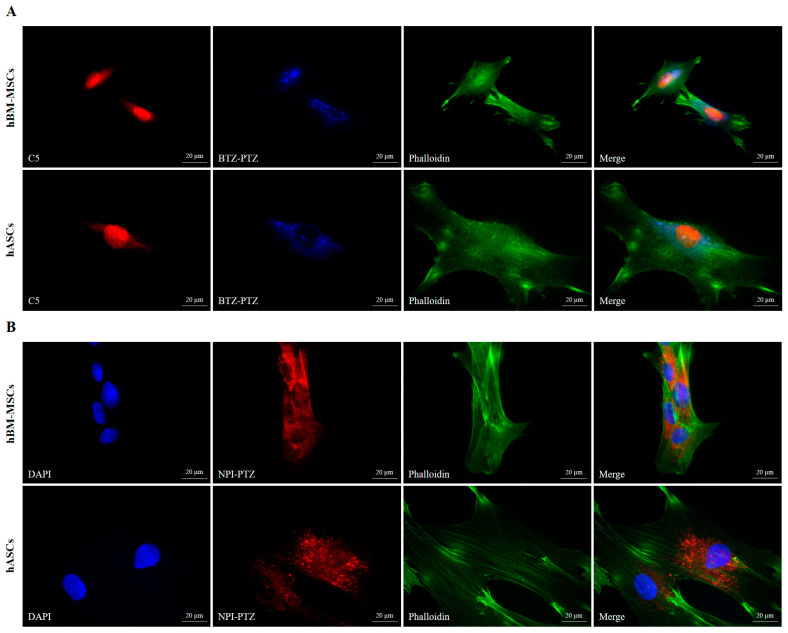
Representative fluorescence microscopy images of fixed hBM-MSCs (upper panels) and hASCs (lower panels). (**A**) Cells stained with 10 μM **BTZ-PTZ** (blue, DAPI filter) and the nuclei marker C5 (red, TRITC filter), along with relative phalloidin (green, FITC filter) and merged images (image magnification: 60×). (**B**) Cells stained with 10 μM **NPI-PTZ** (red, TRITC filter) and the nuclei marker DAPI (blue, DAPI filter), along with relative phalloidin (green, FITC filter) and merged images (image magnification: 60×).

The fluorescence images (Figure 3A,B) illustrate the intracellular distribution of **BTZ-PTZ** and **NPI-PTZ** with a concentration of 10 μM in hBM-MSCs and hASCs. In order to simultaneously visualize the localization of the compounds and the nuclei of the cells, proper nuclear markers were chosen, with the aim being to avoid overlapping with the fluorescence of the studied molecule. For the **BTZ-PTZ** compound (Figure 3A), characterized by blue emissions, the red nuclear dye C5 was used, while in the case of **NPI-PTZ** (Figure 3B), with its red emissions, the DAPI marker was chosen to highlight the nuclei, with its blue fluorescence. As water is a bad solvent for these hydrophobic molecules, both **BTZ-PTZ** and **NPI-PTZ** tend to form aggregates in aqueous environments, leading to a phenomenon known as aggregation-induced emission (AIE). Indeed, this aggregation enhances the fluorescence abilities of the two compounds, whose bright emissions can be recognized in rounded structures, corresponding to the nanoscale aggregates, allowing their intracellular distribution to be easily visualized. Predominant perinuclear localization can be detected for the two compounds. Specifically, **BTZ-PTZ** concentrates near the nucleus. **NPI-PTZ** exhibits a similar distribution, with a predominantly perinuclear signal, although slightly more diffuse within the cytoplasm compared to **BTZ-PTZ**. The specific perinuclear localization of both compounds may reflect a preference for specific subcellular organelles, whose lipid membrane could represent a target for the lipophilic aggregates. These organelles are involved in metabolic and intracellular transport processes, which are extremely relevant to proper cell functioning [49]. Hence, the interaction with the two AIE luminogens may influence their biological activity.

To further investigate the cytoskeletal organization and the potential impact of the compounds on cellular structure, phalloidin, a specific marker for actin filaments [34], was used. Phalloidin staining clearly revealed the architecture of the cytoskeleton, highlighting a well-defined organization of actin filaments in both hBM-MSCs and hASCs. These results confirm that the aggregates of the two compounds, **BTZ-PTZ** and **NPI-PTZ**, selectively accumulate near the nucleus. These findings demonstrate that both **BTZ-PTZ** and **NPI-PTZ** are effectively internalized by both hBM-MSCs and hASCs, highlighting the potential of stem cells as carriers for delivering these compounds to targeted sites.

**Figure 4:**
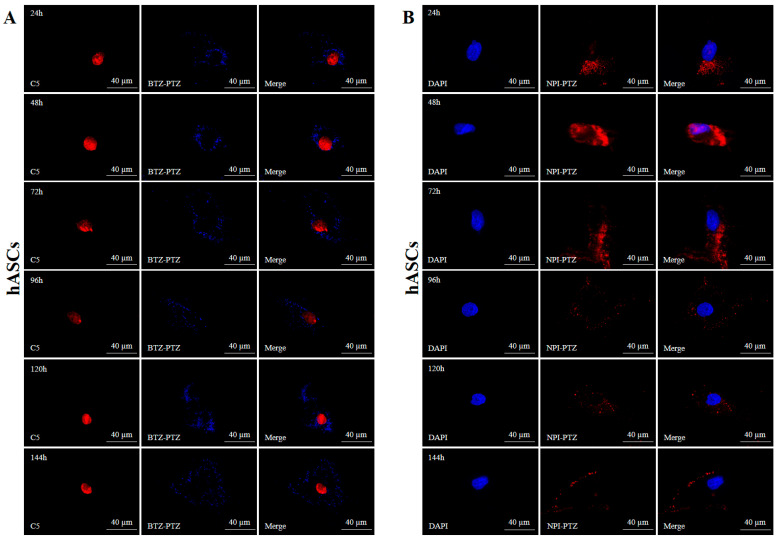

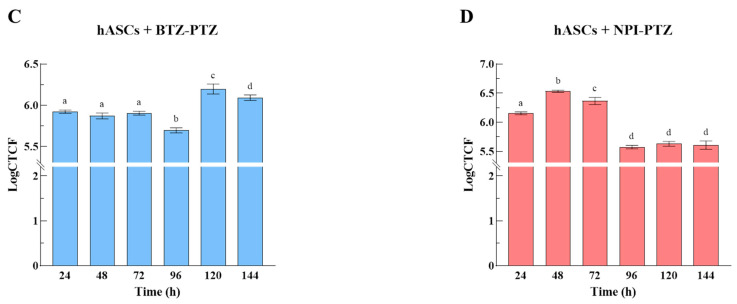
Intracellular fluorescence of **BTZ-PTZ** and **NPI-PTZ** in hASCs over time. Representative fluorescence microscopy images of cells showing the fluorescence signal of 10 µM **BTZ-PTZ** (**A**) and **NPI-PTZ** (**B**) at different time points (24, 48, 72, 96, 120, and 144 h) after internalization (image magnification: 60×). Histograms represent the fluorescence intensity of **BTZ-PTZ** (**C**) and **NPI-PTZ** (**D**), expressed as LogCTCF, as a function of time. Data are presented as mean ± SD (*n* = 5). Columns labeled with different letters are significantly different at *p* < 0.05.

Figure 4 and Figure S3 show the intracellular fluorescence stability of the compounds **BTZ-PTZ** and **NPI-PTZ** in hBM-MSCs and hASCs, respectively, evaluated through fluorescence microscopy over 6 days (144 h) and quantified by plotting the LogCTCF as a function of time. In hBM-MSCs (Figure S3), both compounds exhibited a rather stable intracellular fluorescence. Following internalization, **BTZ-PTZ** and **NPI-PTZ** maintained high and homogeneous fluorescence intensity throughout the observation period, with a significant attenuation only revealed by the end of the monitoring, as revealed by the histograms showing fluorescence intensities, expressed as LogCTCF, as a function of time (Figure S3C,D).

A similar trend was observed in hASCs (Figure 4). In these cells, **BTZ-PTZ** retained a bright and quite stable fluorescence signal throughout the 144 h of observation, whereas **NPI-PTZ** underwent a more apparent decay. The fluorescence of **NPI-PTZ** in hASCs significantly decreased after the first 72 h, with an appreciable loss of signal as shown by the values of LogCTCF (Figure 4D). In addition to this, there was a net change in the distribution of the fluorescence signal over time in hASCs. While in hBM-MSCs, both compounds retained perinuclear localization, in hASCs, the fluorescence signal at long observation times tended to lose its characteristic distribution and to be detected away from the nuclei in a widespread fashion. This might be a sign of some sort of effect exerted by the two compounds on subcellular components, such as organelles or macromolecular structures, likely affecting their functionality. Such behavior would be in line with the decrease in cell viability only detected for hASCs after being exposed to the two molecules for long times (≥72 h).

**Figure 5:**
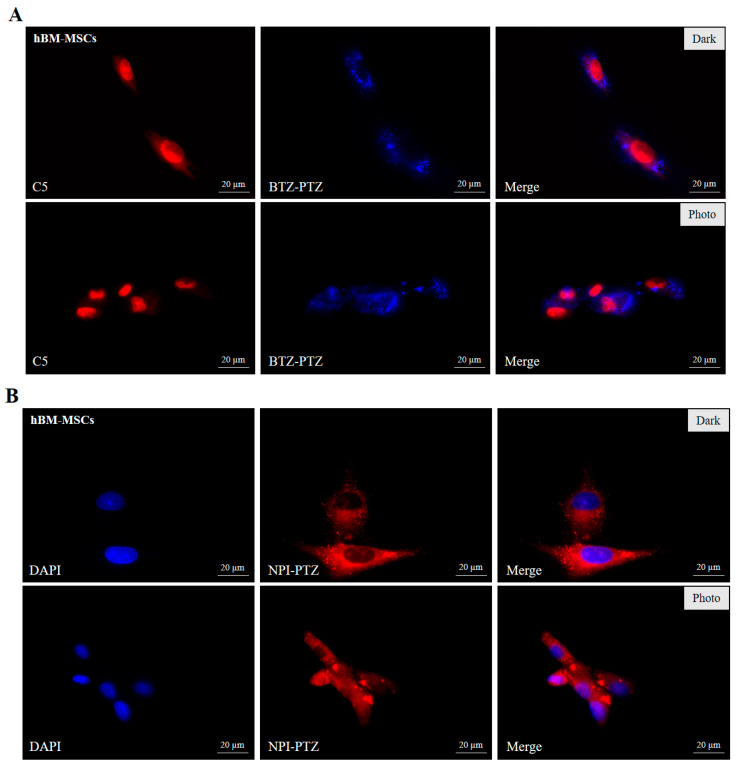
Representative fluorescence microscopy images of 10 µM **BTZ-PTZ** (**A**) and **NPI-PTZ** (**B**) with hBM-MSCs in dark conditions (upper panels) and after 25′ of photoexposure in an LED chamber; (λ_exc_ = 390–400 nm and a power of 1.7 mW/cm^2^) (lower panels). Image magnification: 60×.

**Figure 6:**
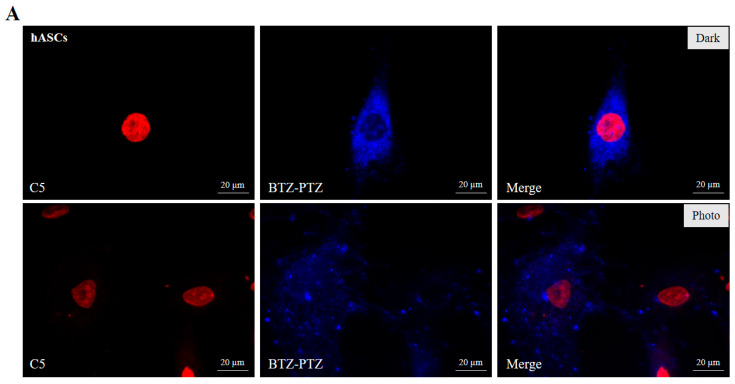

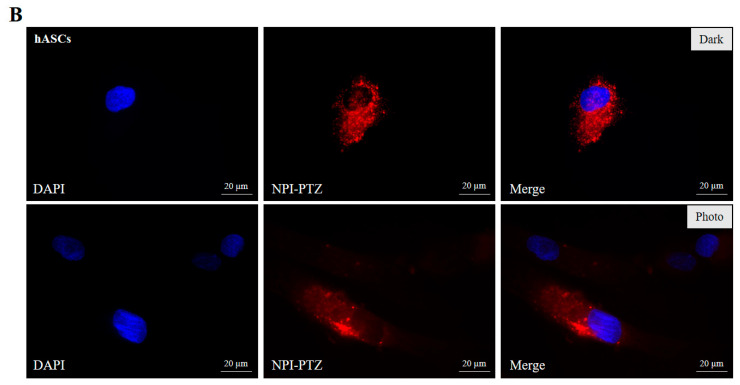
(**A**) Representative fluorescence microscopy images of 10 µM **BTZ-PTZ** (**A**) and **NPI-PTZ** (**B**) with hASCs in dark conditions (upper panels) and after 25′ of photoexposure in an LED chamber; (λ_exc_ = 390–400 nm and a power of 1.7 mW/cm^2^) (lower panels). Image magnification: 60×.

Finally, the effect of light on the treated cells was also investigated by acquiring fluorescence images of the two cell lines after a photoexposure of 25 min in the LED chamber and an incubation of 2 h. Representative results are reported in Figure 5 and Figure 6 for hBM-MSCs and hASCs, respectively. In this case, as a consequence of light exposure, no changes in the fluorescence signal distribution of **NPI-PTZ**, which remained mainly in the perinuclear region, were detected in both cell lines, while the typical perinuclear localization was partially lost for **BTZ-PTZ** in hASCs. Conversely, a clear effect could be recognized on either the dimension and shape of the nuclei or their fluorescence after photoexposure of the two molecules. In particular, in hBM-MSCs, the nuclei tended to become smaller and to acquire a less defined shape relative to their common rounded profile detected in the absence of light. In fact, the mean Feret’s diameter measured along the major axis of the hBM-MSCs nuclei (Figure S4) was reduced to 56% with photoexposure in **NPI-PTZ**-treated cells and to 65% in **BTZ-PTZ**-treated cells. On the contrary, no significant variations were detected for the nuclei of hASCs (Figure S5). Moreover, a slight reduction in the C5 or DAPI fluorescence could be revealed in both cell lines, especially in hASCs (Figures S6 and S7). Both effects highlight how photoexposure takes its toll directly on the nuclei and thus explain the paramount impact of light on cell viability.

## 4. Discussion

In this work, two fluorescent phenothiazine (PTZ) derivatives were incubated within two stem cell lines with the aim being to evaluate the possible application of the two molecules as either fluorescent markers in bioimaging or as sensitizers in stem-cell-driven photodynamic therapy (PDT). Fluorescence images revealed a good internalization of both compounds with a specific and localized distribution in the perinuclear region of the cells (Figure 3). The simultaneous revelation of the fluorescent signal of an appropriate nuclear marker, complementary with the emission of the dyes (DAPI or C5 for **NPI-PTZ** and **BTZ-PTZ**, respectively), and that of phalloidin to stain the cytoskeleton of the cells, highlighted the perinuclear localization of both compounds. Their fluorescence could indeed be easily recognized as bright punctuate structures due to the formation of aggregates with dimensions on the nanometer scale, as is typical of this kind of molecule in an aqueous environment [2,50]. The aggregation of these molecules comes as a result of the lipophilicity of their molecular structure, which may contribute to their localization in subcellular compartments rich in endomembranes, particularly near the nucleus, where the aggregates can be embedded, thus accumulating more intensively [51]. The aggregation is also responsible for the so-called aggregation-induced emission (AIE) phenomenon, which enhances the fluorescent signal of the two markers, making them potentially suitable for bioimaging and imaging-guided treatments. Their fluorescence was also monitored over time (Figure 4 and Figure S3), revealing a good level of stability after up to 6 days of incubation, potentially allowing bioimaging on a long time scale. This behavior parallels a modest effect on cell viability as revealed by cell growth inhibition analysis and the MTT test (Figure 1, Figures S1 and S2). Both experiments indeed demonstrated how hBM-MSC viability is not affected by either any concentration of the two fluorescent probes as high as 10 µM or an increase in the incubation time for as long as 96 h. Conversely, hASCs treated with high concentrations of the two PTZ derivatives showed slight growth inhibition after long incubation times, in line with a loss of perinuclear localization as revealed by fluorescence imaging (Figure 4). This might be the consequence of some sort of specific interaction with subcellular structures involved in metabolic or intracellular transport processes and reveals the higher susceptibility of hASCs. The greater responsiveness of hASCs to both **BTZ-PTZ** and **NPI-PTZ**, even under dark conditions, may be related to their lower intrinsic resistance to oxidative stress. Studies have shown that hASCs display a reduced expression of key antioxidant defense genes such as Nrf2, HO-1, SOD-1, and CAT, particularly when compared to other mesenchymal stem cell subtypes like HC016, which are derived from hASCs via preconditioning with low-dose H_2_O_2_. In particular, hASCs elicit a weaker antioxidant response in oxidatively stressed target cells, with significantly reduced induction of key cytoprotective enzymes [52]. This weakened redox regulation may predispose hASCs to a greater accumulation of ROS and increased susceptibility to cytotoxic stimuli, including non-photoactivated PTZ derivatives. This is further supported by findings indicating that hASCs exhibit a less distinct mechanoresponsive behavior than hBM-MSCs, which may reflect broader limitations in stress adaptation pathways, including those involved in oxidative stress resistance [33]. The results regarding cytotoxicity under dark conditions reported in this study, however, indicate minor effects even on vulnerable stem cells, thus envisaging a plausible employment of the studied PTZ derivatives in long-term bioimaging.

Moreover, the ability of the cells to uptake and retain the compounds, combined with their selective accumulation in a perinuclear region and resistance to intracellular degradation, suggests that these systems could also be further explored for therapeutic applications that require precise compound delivery, as in stem cell-driven PDT. In fact, phototoxicity tests showed an important reduction in cell viability, as demonstrated by the cell growth inhibition curves and IC_50_ values (Figure 1 and Table 1). These analyses disclosed the greater phototoxic effect exerted by **NPI-PTZ** relative to **BTZ-PTZ** and the higher vulnerability of hASCs relative to hBM-MSCs. IC_50_ values were found in the micromolar range, with the lowest value (1.3 ± 0.1 µM) measured for the combination of hASCs treated with photoactive **NPI-PTZ**. Cell growth inhibition curves corroborated these data, giving insights into the mechanism of action of the two photosensitizers as a function of time: once photoexposed, the two PTZ compounds were responsible for a reduction in hBM-MSC viability, which reached a minimum within 48 h, finally returning to higher values at the latest observation time point; conversely, when it comes to hASCs, in addition to the substantial initial drop in cell viability, a further reduction was also recorded at later time points as an effect of the intrinsic toxicity of the two compounds for this cell line. As further proof of light-induced toxicity, fluorescence images of photoexposed cells (Figure 5 and Figure 6) showed modifications of the nuclear features in terms of either size and shape or fluorescence intensity.

All of these findings suggest two possible outcomes brought about by the interaction of the two molecules with stem cells: one is mild dark cytotoxicity and the other is triggered by light. On the one hand, the decrease in cell viability in the dark, only measured for hASCs after long incubation times, might rely on the effect exerted by the two dyes on the functionality of those organelles they are bound to; on the other hand, light triggers a specific and immediate response, supposedly related to ROS production, that induces cell death by directly affecting the nuclei. In fact, upon photoexcitation, both molecules are known to undergo intersystem crossing to a triplet state, which could promote energy transfer to nearby molecules, such as molecular oxygen to produce singlet oxygen, as already found for both compounds in solution and, in the case of **BTZ-PTZ**, also in tumor cells [2,31]. For a straightforward assignment of the compounds’ phototoxic effect to the production of ROS, including in stem cells, their intracellular levels in photoexposed cells treated with the two molecules were measured and found to be high above their standard values detected in untreated cells (Figure 2). This result is extremely alluring in light of stem cell-driven PDT, because ROS production, in addition to directly compromising cellular structures such as mitochondria and nuclei, can also trigger extracellular signaling, leading to a bystander effect, as extensively described in the literature [37,53,54,55,56]. This phenomenon occurs when stressed or apoptotic cells release signaling molecules such as cytokines, damage-associated molecular patterns (DAMPs), or additional ROS, which propagate the death signal to neighboring cells. Consequently, the local cellular environment is further compromised, amplifying the cytotoxic response beyond the initially targeted cells [37,56]. In this way, not only can the two photosensitizers exert a phototoxic effect on the stem-cell vehicles, but also targeted tumor cells can in turn be damaged. Hence, given their stable and bright emissions and their toxicity, especially controlled by light, the two molecules become promising photosensitizers for their possible future employment in fluorescence-guided stem cell-driven PDT.

## 5. Conclusions

This study reports two phenothiazine (PTZ) derivatives substituted with a naphthalimide (NPI) and a benzothiazole (BTZ) as possible fluorescent probes for bioimaging and photosensitizers for stem cell-driven PDT. By exploiting their AIE behavior, the fluorescence of the two compounds was detected after incubation within two stem cell lines, namely hBM-MSCs and hASCs, where the molecular nanoaggregates were found to specifically localize in the perinuclear region of the cells, as a plausible consequence of membrane-targeted delivery. Their fluorescence proved to be stable for days, allowing the fate of the fluorescent markers to be monitored for up to six days, while not causing major cytotoxic effects on the cells. On the other hand, when triggered by light, the two photosensitizers induced significant inhibition of cell growth, thus exerting high phototoxicity as a consequence of relevant ROS production. Therefore, by combining the ability of stem cells to naturally migrate towards tumor sites, the remarkable and specific emissions of the two AIE luminogens, and the phototoxic action that the latter could induce in both the recipient cells and the surrounding environment, an application of the two molecules in fluorescence-guided stem cell-driven PDT can likely be envisaged.

## Figures and Tables

**Table 1:** Stem cell growth inhibition by **BTZ-PTZ** and **NPI-PTZ** presented as IC_50_ values (in μM) ^a^ upon different durations of light irradiation (12′ 30″ and 25′ in an LED chamber with λ_exc_ = 390–400 nm and a power of 1.7 mW/cm^2^) evaluated through the MTT test after 72 h of incubation and expressed as the mean of two independent experiments of four replicas each ± SD.

Cells	IC_50/_µM			
	BTZ-PTZ		NPI-PTZ	
	12′ 30″	25′	12′ 30″	25′
**hBM-MSCs**	-	8.1 ± 0.9	10 ± 1	6.3 ± 0.6
**hASCs**	>10	2.2 ± 0.2	6.6 ± 0.7	1.3 ± 0.1

^a^ IC_50_: concentration that causes 50% cell viability inhibition.

## Data Availability

Dataset available on request from the authors.

## Supplementary Materials

The following supporting information can be downloaded at: https://www.mdpi.com/article/10.3390/nano15120894/s1, Figure S1: Antiproliferative effect on stem cells kept in the dark; Figure S2: Antiproliferative effect on photoexposed stem cells; Figure S3: Intracellular fluorescence in hBM-MSCs over time; Figures S4 and S5: Maximum Feret’s diameter of nuclei of stem cells kept in the dark and photoexposed;. Figures S6 and S7: Fluorescence intensity of nuclei of stem cells kept in the dark and photoexposed.
