# Supplementary material for: Phenothiazine-Based Nanoaggregates: Dual Role in Bioimaging and Stem Cell-Driven Photodynamic Therapy
Source: Nanomaterials (Basel). 2025 Jun 10;15(12):894. doi: 10.3390/nano15120894 (PMC12196274; doi:10.3390/nano15120894)
Supplement: Supplementary file 1 [file nanomaterials-15-00894-s001.zip › nanomaterials-3626059-supplementary.pdf]

nanomaterials

MDPI

Article

Phenothiazine-Based Nanoaggregates: Dual Role in Bioimaging and Stem Cell-Driven Photodynamic Therapy

Eleonora Calzoni [1], Alessio Cesaretti [1,2,*], Nicolò Montegiove [3], Maria Luisa Valicenti [1], Francesco Morena [1], Rajneesh Misra [4], Benedetta Carlotti [1,2] and Sabata Martino [1,2]

[1] Department of Chemistry, Biology and Biotechnology, University of Perugia, 06100 Perugia, Italy; eleonora.calzoni@unipg.it (E.C.); marialuisa.valicenti@dottorandi.unipg.it (M.L.V.); francesco.morena@unipg.it (F.M.); benedetta.carlotti@unipg.it (B.C.); sabata.martino@unipg.it (S.M.)
[2] Centro di Eccellenza Materiali Innovativi Nanostrutturati (CEMIN), University of Perugia, via Elce di Sotto 8, 06123 Perugia, Italy
[3] Department of Civil and Environmental Engineering, University of Perugia, Via G. Duranti 93, 06125 Perugia, Italy; nicolo.montegiove@unipg.it
[4] Department of Chemistry, Indian Institute of Technology Indore, Indore 453552, India; rajneeshmisra@iiti.ac.in
* Correspondence: alessio.cesaretti@unipg.it

Supporting Information

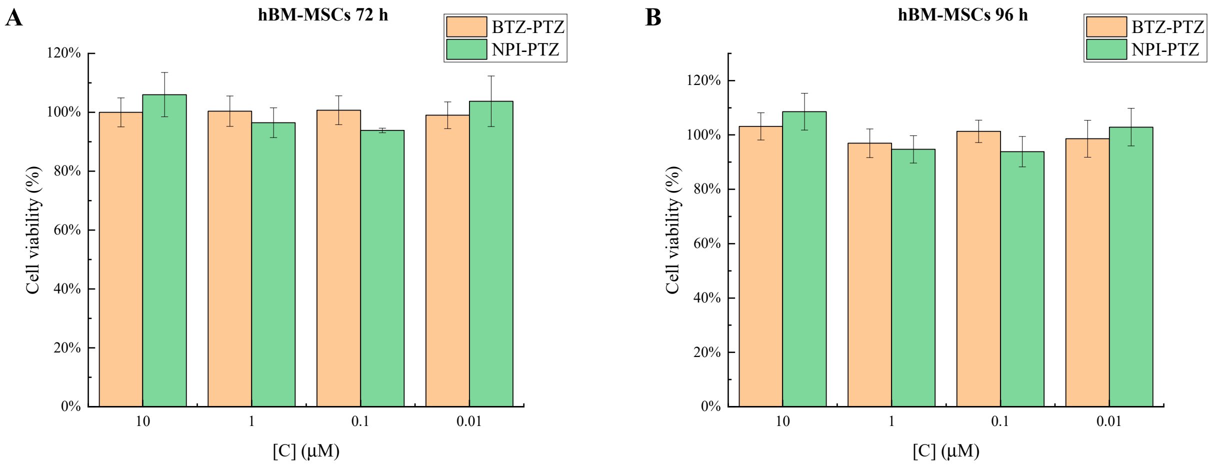

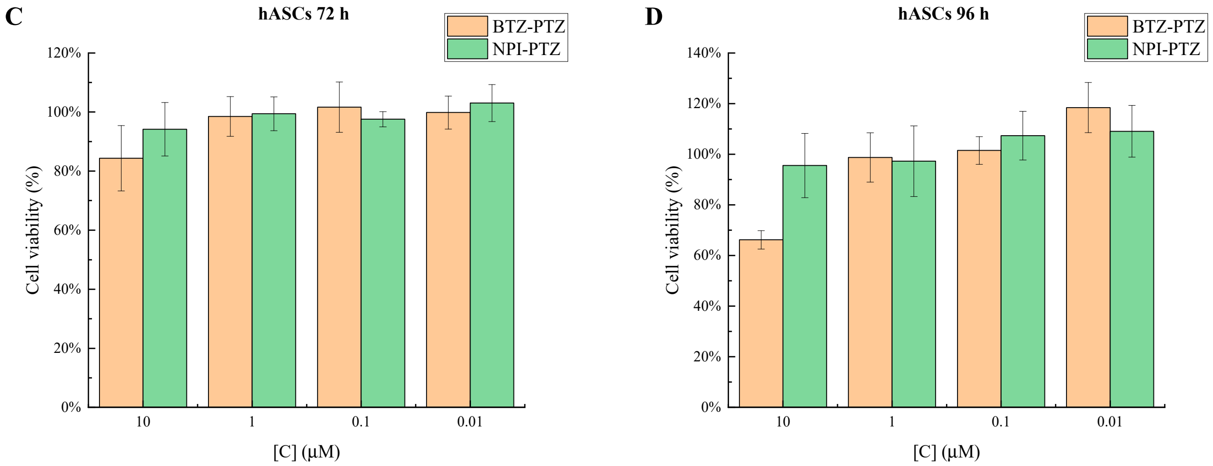

Figure S1. Antiproliferative effect exerted by compounds **BTZ-PTZ** and **NPI-PTZ** on hBM-MSCs (upper panels) and hASCs (lower panels) kept in the dark as evaluated from the MTT test after an incubation of 72 (left) or 96 (right) hours and expressed as the mean of two independent experiments of four replicas each ± SD; 100% corresponds to control mean values.

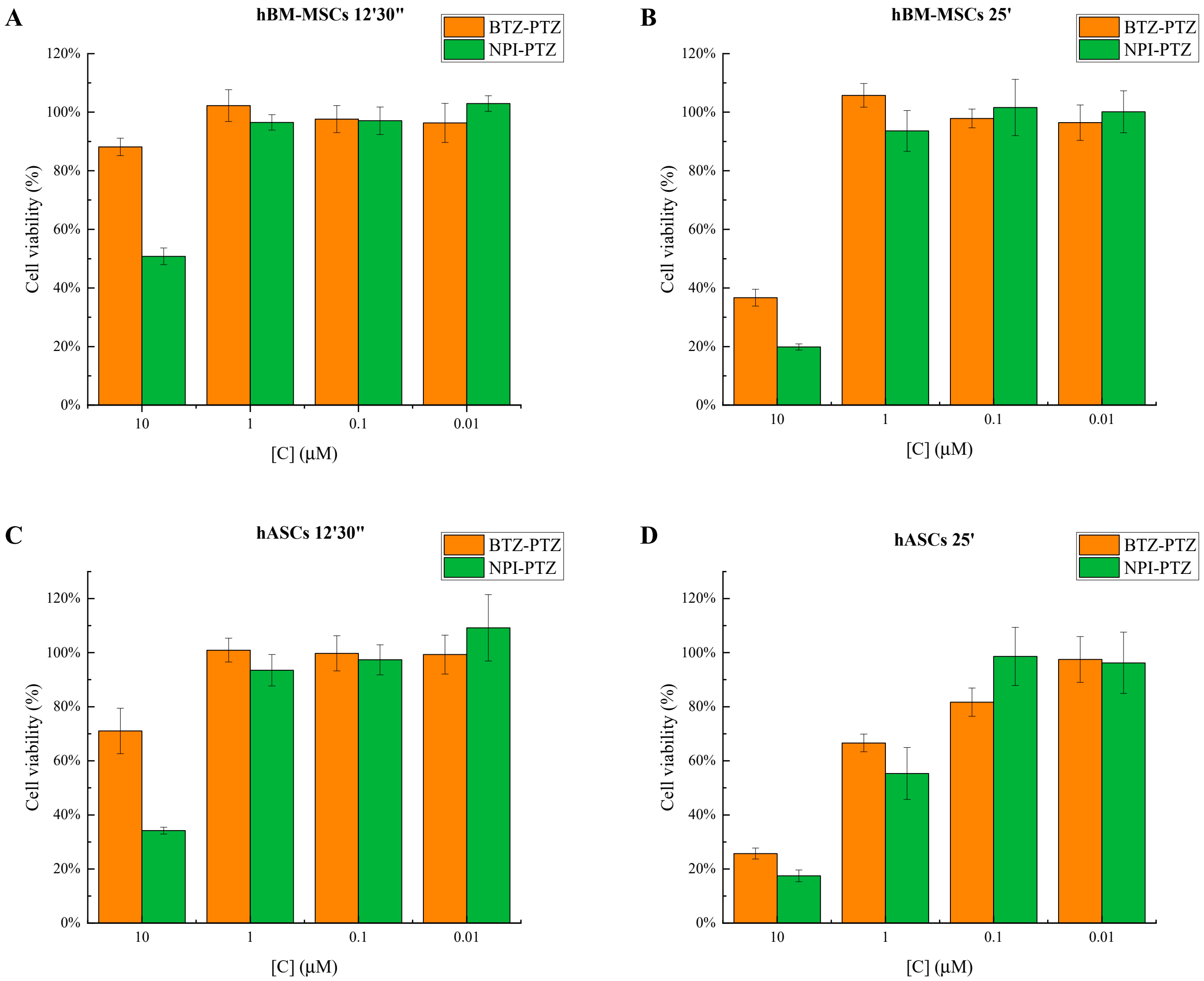

Figure S2. Antiproliferative effect exerted by compounds **BTZ-PTZ** and **NPI-PTZ** on hBM-MSCs (upper panels) and hASCs (lower panels) photoexposed for 12′ 30″ (left) and 25′ (right) in an LED chamber (λ_{exc} = 390–400 nm and a power of 1.7 mW/cm^2) as evaluated from the MTT test after 72-hour incubation and expressed as the mean of two independent experiments of four replicas each ± SD; 100% corresponds to control mean values.

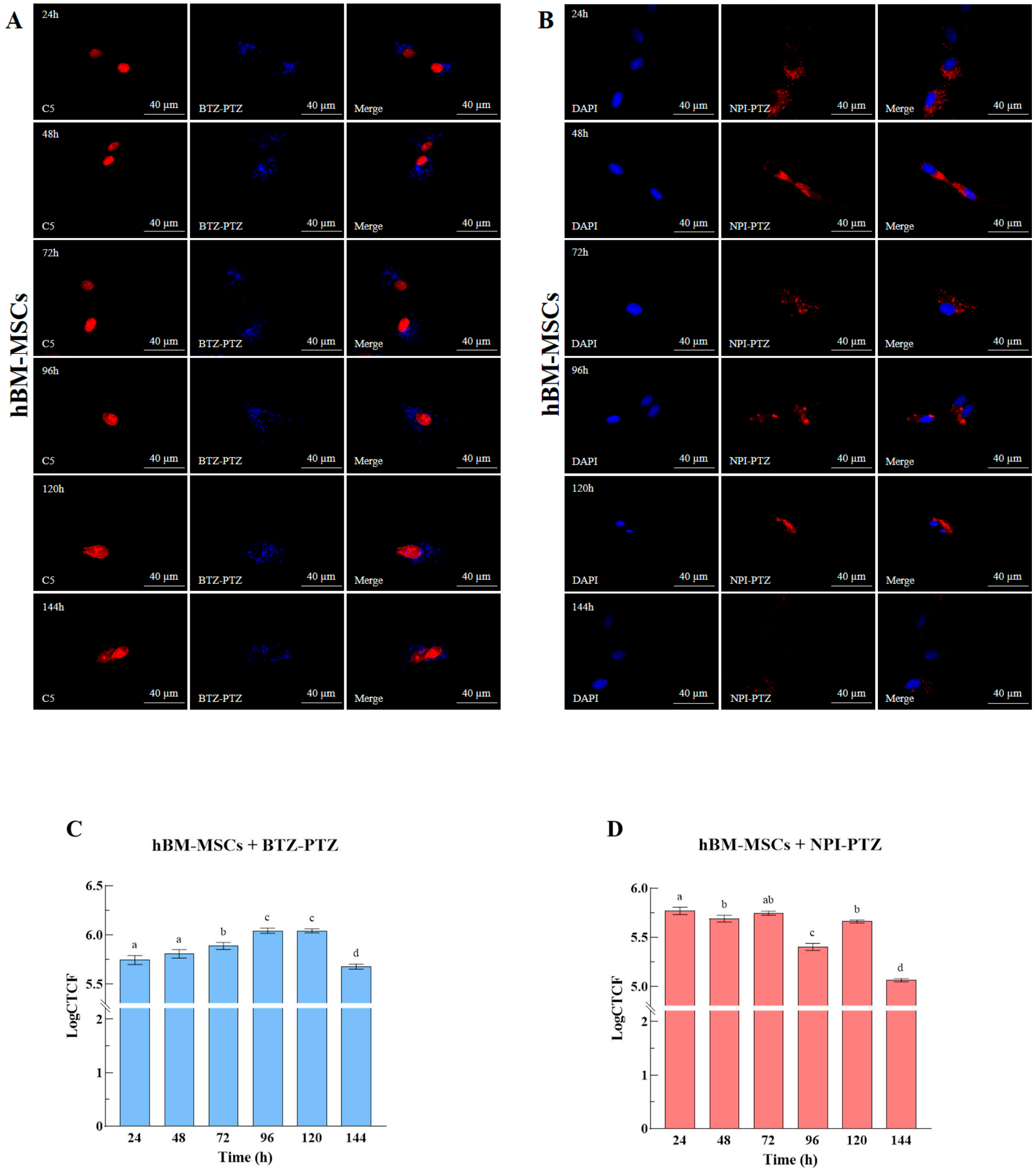

Figure S3. Intracellular fluorescence of **BTZ-PTZ** and **NPI-PTZ** in hBM-MSCs over time. Representative fluorescence microscopy images of cells showing the fluorescence signal of 10 µM **BTZ-PTZ** (**A**) and **NPI-PTZ** (**B**) at different time points (24, 48, 72, 96, 120, and 144 hours) after internalization (image magnification: 60×). Histograms represent the fluorescence intensity of **BTZ-PTZ** (**C**) and **NPI-PTZ** (**D**), expressed as LogCTCF, as a function of time. Data are presented as mean ± SD ($n = 5$). Columns labeled with different letters are significantly different at $p < 0.05$.

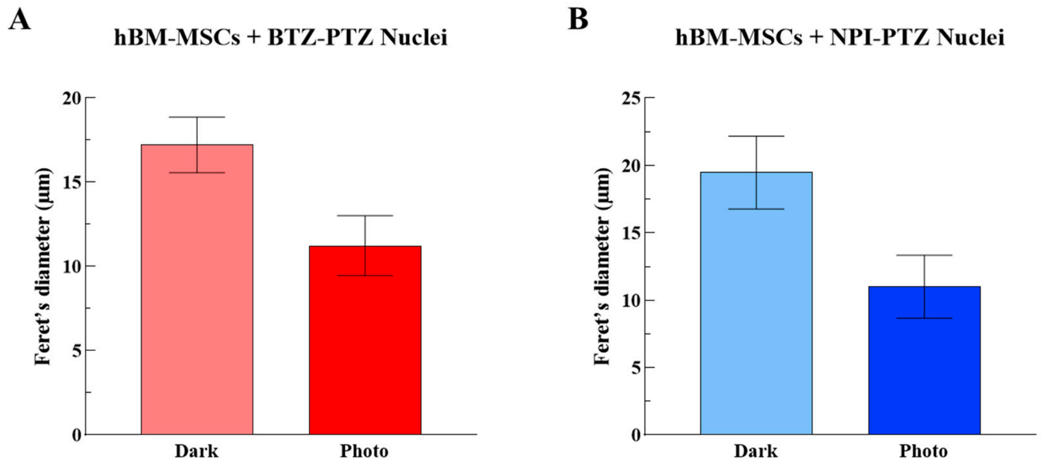

Figure S4. Mean maximum Feret's diameter ± SD of nuclei of hBM-MSCs treated with **BTZ-PTZ** (**A**) and **NPI-PTZ** (**B**) in dark conditions and after 25′ of photoexposure in an LED chamber (λ_{exc} = 390–400 nm and a power of 1.7 mW/cm^2).

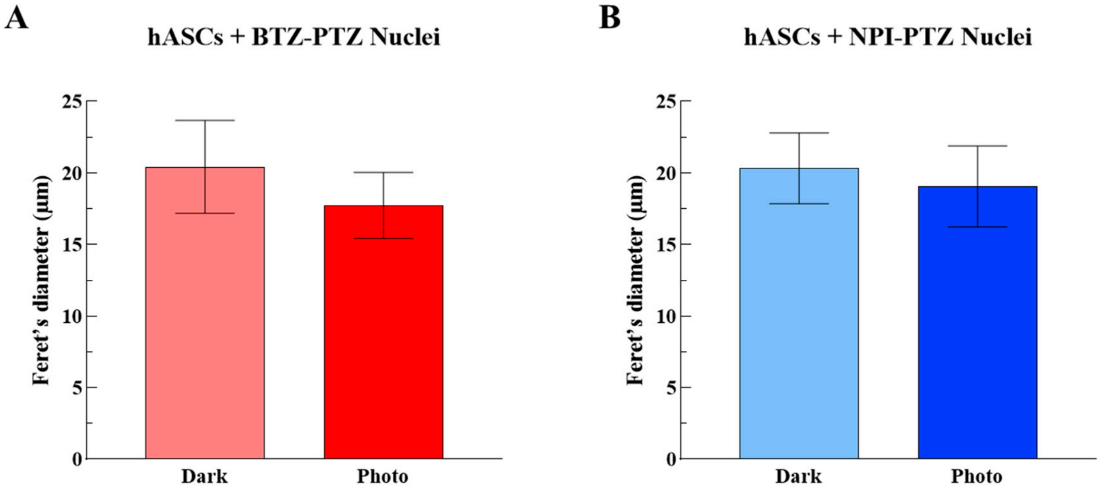

Figure S5. Mean maximum Feret's diameter ± SD of nuclei of hASCs treated with **BTZ-PTZ** (**A**) and **NPI-PTZ** (**B**) in dark conditions and after 25′ of photoexposure in an LED chamber (λ_{exc} = 390–400 nm and a power of 1.7 mW/cm^2).

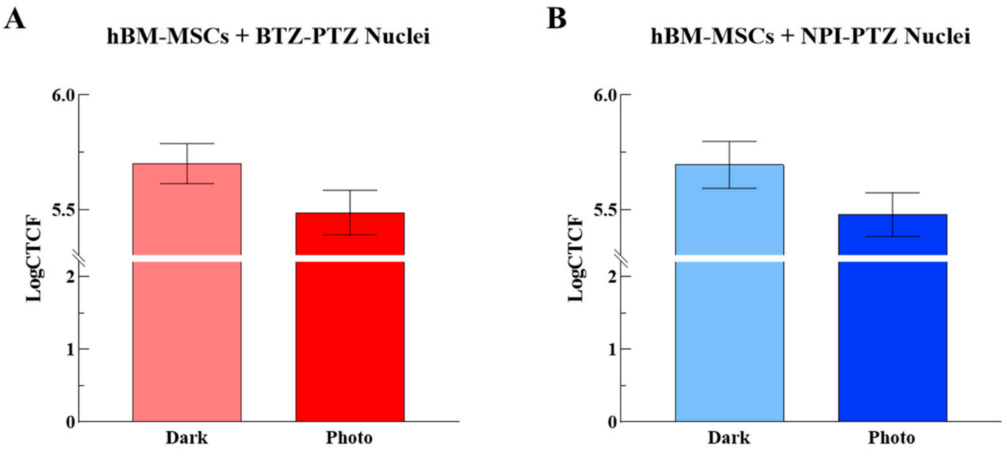

Figure S6. Mean fluorescence intensity ± SD of nuclei of hBM-MSCs treated with **BTZ-PTZ** (**A**) and **NPI-PTZ** (**B**) in dark conditions and after 25′ of photoexposure in an LED chamber (λ_{exc} = 390–400 nm and a power of 1.7 mW/cm^2).

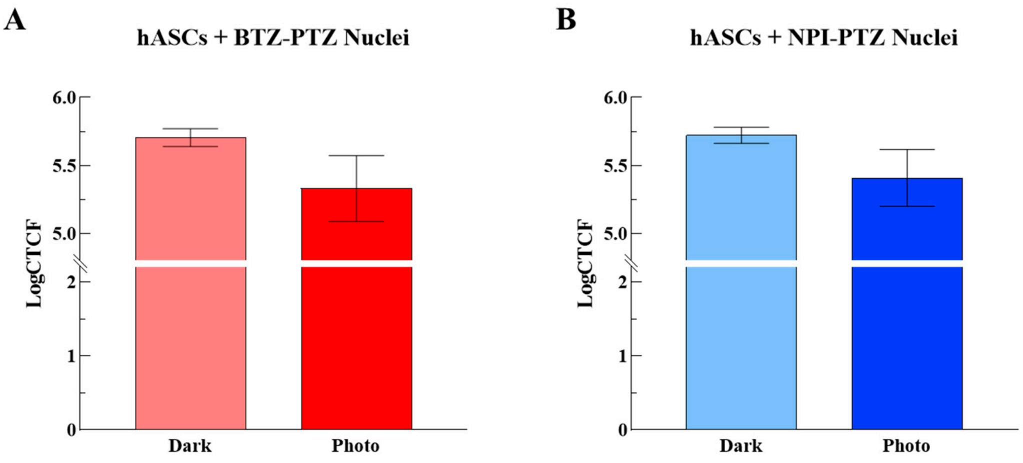

Figure S7. Mean fluorescence intensity ± SD of nuclei of hASCs treated with **BTZ-PTZ** (**A**) and **NPI-PTZ** (**B**) in dark conditions and after 25′ of photoexposure in an LED chamber (λ_{exc} = 390–400 nm and a power of 1.7 mW/cm^2).
